# Identification of Brain Electrical Activity Related to Head Yaw Rotations

**DOI:** 10.3390/s21103345

**Published:** 2021-05-11

**Authors:** Enrico Zero, Chiara Bersani, Roberto Sacile

**Affiliations:** Department of Informatics, Bioengineering, Robotics, and Systems Engineering (DIBRIS), University of Genova, 16145 Genoa, Italy; chiara.bersani@unige.it (C.B.); roberto.sacile@unige.it (R.S.)

**Keywords:** brain-computer interface, system identification, feedforward neural networks, brain electrical activity

## Abstract

Automatizing the identification of human brain stimuli during head movements could lead towards a significant step forward for human computer interaction (HCI), with important applications for severely impaired people and for robotics. In this paper, a neural network-based identification technique is presented to recognize, by EEG signals, the participant’s head yaw rotations when they are subjected to visual stimulus. The goal is to identify an input-output function between the brain electrical activity and the head movement triggered by switching on/off a light on the participant’s left/right hand side. This identification process is based on “Levenberg–Marquardt” backpropagation algorithm. The results obtained on ten participants, spanning more than two hours of experiments, show the ability of the proposed approach in identifying the brain electrical stimulus associate with head turning. A first analysis is computed to the EEG signals associated to each experiment for each participant. The accuracy of prediction is demonstrated by a significant correlation between training and test trials of the same file, which, in the best case, reaches value *r* = 0.98 with *MSE* = 0.02. In a second analysis, the input output function trained on the EEG signals of one participant is tested on the EEG signals by other participants. In this case, the low correlation coefficient values demonstrated that the classifier performances decreases when it is trained and tested on different subjects.

## 1. Introduction

In human computer interaction (HCI), design and application of brain–computer interfaces (BCIs) are among the main challenging research activities. BCI technologies aim at converting human mental activities into electrical brain signals, producing a control command feedback to external devices such as robot systems [1]. Recently, scientific literature has shown specific interest in cognitive human reactions’ identification, caused by a specific environment perception or an adaptive HCI [2]. Reviews on BCI and HCI can be found in Mühl et al. [3] and Tan and Nijholt [4].

The essential stages for a BCI application consist of a signal acquisition of the brain activities, on the preprocessing and feature extraction, classification, and feedback.

The brain signals acquisition may be realized by different devices such as Electroencephalography (EEG), Magnetoencephalography (MEG), Electrocorticography (ECoG), or functional near infrared spectroscopy (fNIRS) [5]. The preprocessing consists of cleaning the input data from noises (called artifacts), while the extraction feature phase deals with selecting, from the input signals, the most relevant features required to discriminate the data according to the specific classification [6]. The classification is the central element of the BCI and it refers to the identification of the correct translation algorithm, which converts the extracting signals features into control commands for the devices according to the user’s intention.

From the signals acquisition viewpoint, the EEG represents the most used technique; although it is non-invasive, cheap, and portable, it assures a good spatial and temporal resolution [7]. As stated in the literature [8], however, the acquisition of EEG signals through hair remains a critical issue.

Electroencephalography (EEG)-based biometric recognition systems have been used in a large range of clinical and research applications [9] such as interpreting humans’ emotional states [10], monitoring participants’ alertness or fatigue [11], checking memory workload [12], evaluating participants’ fear when subjected to unpredictable acoustic or visual external stimuli [13], or diagnosing generic brain disorders [14].

Significant literature concerning EEG signal analysis versus visual-motor tasks is available. Perspectives are particularly relevant in rehabilitation engineering. A pertinent recent example of EEG analysis application in robotics and rehabilitation engineering is provided in Randazzo et al. [15], where the authors tested on nine participants how an exoskeleton, coupled with a BCI, can elicit EEG brain patterns typical of natural hand motions.

Apart from this, cognitive activities related to motor movements have been observed in EEG following both actually executed and imagined actions [16,17]. Comparing neural signals provided by actual or imaginary movements, most papers concluded that the brain activities are similar [18]. In literature, a significant correlation between head movements and visual stimuli has been proven [19].

In order to realize an EEG-based BCI, adopting a classifier to interpret EEG-signals and implement a control system is necessary. In fact, according to the recorded EEG pattern and the classification phase, EEG may be used as input for the control interface in order to command external devices. As demonstrated in literature, the quality of the classifier, which has to extract the meaningful data from the brain signals, represents the crucial point to obtain a robust BCI [20].

The well-known techniques used for EEG signals classification in motor-imagery BCI applications are support vector machine (SVM), linear discriminant analysis (LDA), multi-layer perceptron (MLP), and random forest (RF) or convolutional neural network (CNN) classifiers. In Narayan [21], SVM obtained better performances with 98.8% classification accuracy in respect to LDA and MLP for left-hand and right-hand movements recognition. In their research, the authors demonstrated the superiority of the CNN in respect to LDA and RF for the classification of different fine hand movements [22]. In Antoniou et al. [23], the RF algorithm outperformed compared to K-NN, MLP, and SVM in the classification of eye movements used in a EEG-based control system for driving an electromechanical wheelchair. In Zero et al. [24], a time delay neural network (TDNN) classification model has been implemented to classify the human’s EEG signals when the driver has to rotate the steering wheel to perform a right or a left turn during a driving task in a simulated environment.

More in general, almost 25% of recent classification algorithms for neural cortical recording were based on Artificial Neural Networks (ANNs) [25], as they have been intensively applied to EEG classification [26]. An interesting application is presented in Craig and Nguyen [27], where the authors proposed an ANN classifier for mental command with the purpose of enhancing the control of a head-movement controlled power wheelchair for patients with chronic spinal cord injury. The authors obtained an average accuracy rate of 82.4%, but they also noticed that the classifier applied to a new subject performed worse than expected and that the customization of the classifier by an adaptive training technique increased the quality of prediction. Besides, researchers used ANNs for motor imagery classification of hand [28] or foot movements [29], as well as eye blinking detection [30]. In Lotte et al. [31], a review on classification algorithms for EEG-based BCI appears.

This paper focuses on an original objective in the context of EEG signals classifiers in respect to the literature related to body movements. Even if this work adopts a traditional ANN classifier, the scope of the application represents the main novelty due to the fact that we explore the recognition of the yaw head rotations directed toward a light target by EEG brain activities to support the driving of tasks in different applications, such as to control autonomous vehicle or wheelchair or robot in general.

In detail, this work is about “using brain electrical activities to recognize head movements in human subjects.” Input data are EEG signals collected from a set of 10 participants. Left or right head position as responses to external visual stimulus represent the output data for the experiments. The main purpose of the proposed approach is defining and verifying the BCI system effectiveness in identifying an input-output function between EEG and head different positions. Section 2 introduces BCI architecture used for experiments, while Section 3 shows results coming from different training and testing scenarios. Section 4 briefly reports the conclusions.

## 2. Materials and Methods

### 2.1. System Architecture

The architecture of the system used for the experiments consists of two interacting sub-systems: (1) a basic lamp system in charge of generating visual stimuli, and (2) an Enobio^®^ EEG systems cap by Neuroelectrics (Cambridge, MA, USA) for EEG signal acquisition. The two subsystems can communicate with a PC server through a serial port and a Bluetooth connector, respectively.

#### 2.1.1. Lamp System

**Figure 1:**
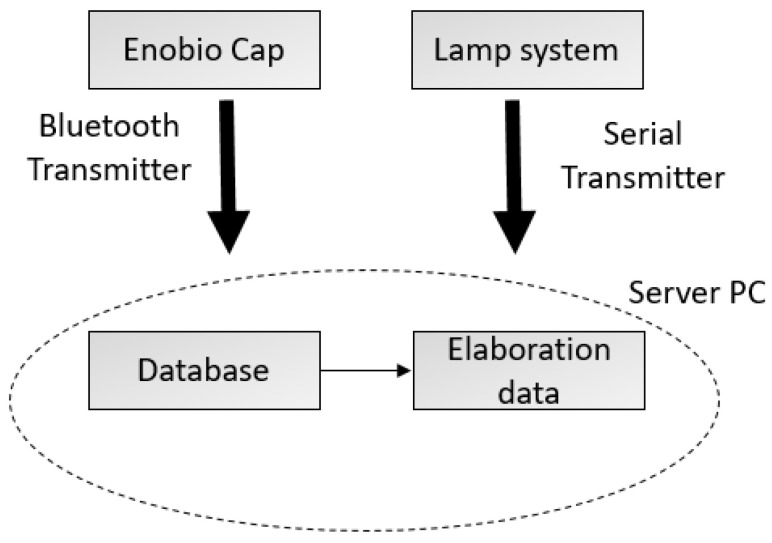
System architecture.

The lamp system’s main components are a Raspberry pi 3-control unit (Cambridge, UK) and two LED lamps. The PC server hosts a Python application, which randomly sends an input to the Raspberry unit by the serial cable. The Raspberry unit hosts another Python application, which receives commands to switch on/off the lamps. Figure 1 shows the system architecture.

**Figure 2:**
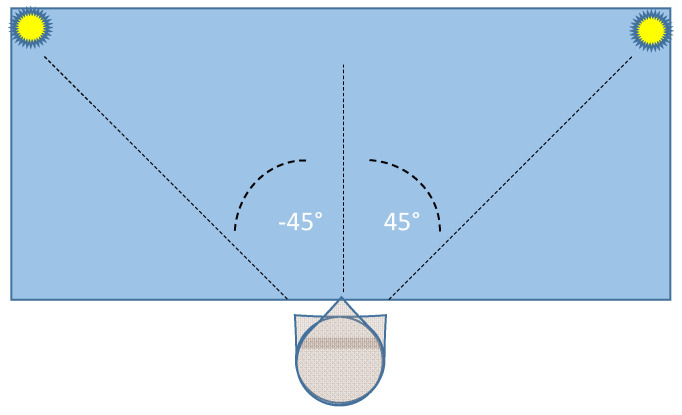
Top vision of the layout of the experimental set environment.

The two lamps are positioned at the extreme sides of a table (size: 1.3 × 0.6 m), allowing a typical head rotation (yaw angle) over a −45°/45° range. Figure 2 shows a top vision of the experimental set environment.

#### 2.1.2. EEG Enobio Cap

**Figure 3:**
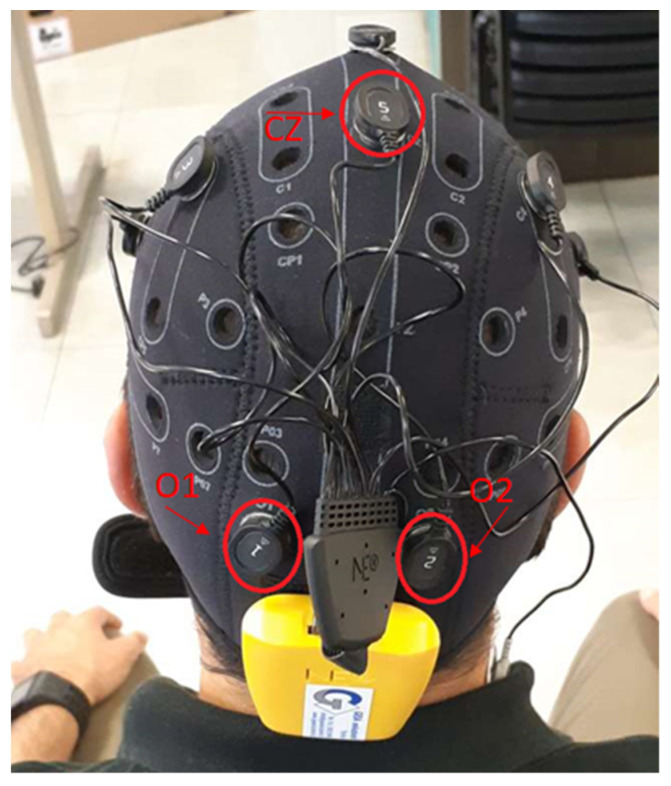
EEG Enobio Cap.

The sensors connected to this cap can monitor EEG signals at 500 Hz frequency. The Enobio cap works on eight different channels. In order to decrease the artifacts due to muscular activity, the EEG system is equipped with two additional electrodes to apply a differential filtering to the EEG signals. These two electrodes are positioned in a hairless area in the head (usually behind the ears by the neck). In the proposed experiments, we focus on three channels labeled O1, O2, and CZ, according to International Standard System 10/20. The first two are positioned in the occipital lobe; the other in the parietal one (Figure 3). The reason for this choice is that the signals coming from the occipital lobe are commonly associated with visual processing [32], while the signals coming from the parietal lobe are related to body movement activities. In addition, a good correlation between occipital centroparietal areas improves visual motor performance identification [33].

Positioning electrodes on the head plays a fundamental role in the quality of the data acquisition. For example, using gel may improve the quality of EEG signal. However, the main target of this work is verifying EEG monitoring’s feasibility in working conditions, in order to avoid every possible, although limited, action on the workers. For this reason, no gel was used in the sensors positioning phase.

### 2.2. Simulation Description

During data acquisition, the participant sits in front of the table and wears the EEG Enobio cap, assisted by the operator who checks the electrodes position. Each participant is expected to move his/her head left or right towards the lamp, which is randomly switched on by the Raspberry unit. The lamp stays on for a variable period of time (between six and nine seconds). After turning off, the lamp stays inactive for five seconds. The test participant is expected to move his or her head back to the starting position following the lamp turning off.

### 2.3. Data Processing and Analysis

#### 2.3.1. Pre-Processing Data

During EEG monitoring, the presence of artifacts and noise in the acquired data was one of the main problems we had to face. Exogenous and endogenous noises can significantly affect reliability of the acquired data. Concerning artifacts, several types have been described in literature [34], among others, such as ocular, muscle, cardiac, and extrinsic artifacts.

In order to limit artifacts, we worked as follows:

- Muscle artifacts were intrinsically limited in the EEG signal acquisition system thanks to the two differential electrodes embodied in Enobio Cap.
- Extrinsic artifacts were limited by proper signal filtering and normalizing EEG signals. Specifically, we applied a bandpass filtering between 49 and 51 Hz in order to eliminate the noise given by the electrical frequencies [35].
- In addition, in order to remove linear trends, a high pass filter—cutting frequencies lower than 1 Hz—filtered the overall signal.

The resulting signals, whose unit of measure is µV, have been amplified to a factor 10^5^, and limited between 1 and −1. The reason is to enhance the precision of the following signal analysis. The head positions were classified as follows: −1 for left position, 1 for right, and 0 for forward. The participants were asked to move the head in a normal speed avoiding sudden movements. Thus, transition from one position to the other (e.g., left to forward) was linearly smoothed using a moving average computed on a window of 300 samples (i.e., for a duration of 0.6 s).

#### 2.3.2. Input Output Data Analysis

The testing goal is to find a direct input-output function that is able to relate a certain number of EEG samples to the related value of the head position. This is challenging since, as stated in literature, time variance [36] and sensibility to different participants’ reactions [37] are well known obstacles.

Specifically, the goal is to identify a non-linear input-output function, which takes 10 consecutive EEG samples, extracted from O1, O2, and Cz, (hereinafter defined as $\underline{x}(t$), which is a 3-component vector sampled at instant t), and the value of the head position in the sample just following the EEG samples (hereinafter defined as y(t)).

A non-linear function f between input $\underline{x}\left(t\right)$ and output y(t) must be identified so that the values $\tilde{y}\left(t\right)$ resulting by Equation (1):

(1) $$\tilde{y}\left(t\right)=f\left(\underline{x}\left(t-1\right),\underline{x}\left(t-2\right),\ldots \underline{x}\left(t-10\right)\right)$$

minimize the minimum squared error (*MSE*) between y(t) and $\tilde{y}\left(t\right)$ values, where *MSE* computed on one prediction is given by:

(2) $$MSE=\frac{\sum_{t=1}^{n}{\left(y\left(t\right)-\tilde{y}\left(t\right)\right)}^{2}}{n}$$

To keep predictions less sensible to the input noise, the predicted values $\tilde{y}\left(t\right)$ are averaged on a moving mean of 300 preceding samples, which is:

(3) $$\bar{y}\left(t\right)=\frac{\sum_{\hat{t}=0}^{299}\tilde{y}\left(t-\hat{t}\right)}{300}$$

Results related to the identification reliability of the function f are evaluated against two key performance indexes, *MSE* and Pearson correlation coefficient *r*, as reported below:

(4) $$MSE\left(y,\bar{y}\right)=\frac{\sum_{t=1}^{n}{\left(y\left(t\right)-\bar{y}\left(t\right)\right)}^{2}}{n}$$

(5) $$r\left(y,\bar{y}\right)=\frac{cov\left(y,\bar{y}\right)}{\sigma_{y}\sigma_{\bar{y}}}$$

where:

- $\sigma_{y}$ and $\sigma_{\bar{y}}$ are the standard deviations of y and $\bar{y}$;
- $cov\left(y,\bar{y}\right)$ is the covariance of y and $\bar{y}$.

An ANN with 10 neurons in the hidden layer identified the non-linear input-output function. The identification process is based on Levenberg–Marquardt backpropagation algorithm [38,39] Matlab^®^ software version R2020 b (Natick, MA, USA). The training processes got a solution after an average of 87 steps (about 45 s) on a common Dell laptop Intel i5-3360M CPU, 2.8 GHz, 8 GB (Austin, TX, USA).

## 3. Results

### 3.1. Data Set

The trials involved 10 participants: one woman (P1) and nine men (P2–P10), aged 25 to 60, with no known history of neurological abnormalities.

All participants, but P5, are right-handed. P2 and P4 are hairless. For two participants, namely P1 and P2, 10 different experiments were recorded; for P10, 2 experiments were recorded while for the others, namely P3–P9, only one experiment was recorded. All tests were 5 min long. Table 1 shows the main files characteristics.

From left to right, the columns show: participant ID; file ID; the number of samples in each file; time elapsed from participant’s first trial; occurrences percentage related to the three coded head positions (1 R (right), 0 F (forward), and −1 L (left)).

**Figure 4:**
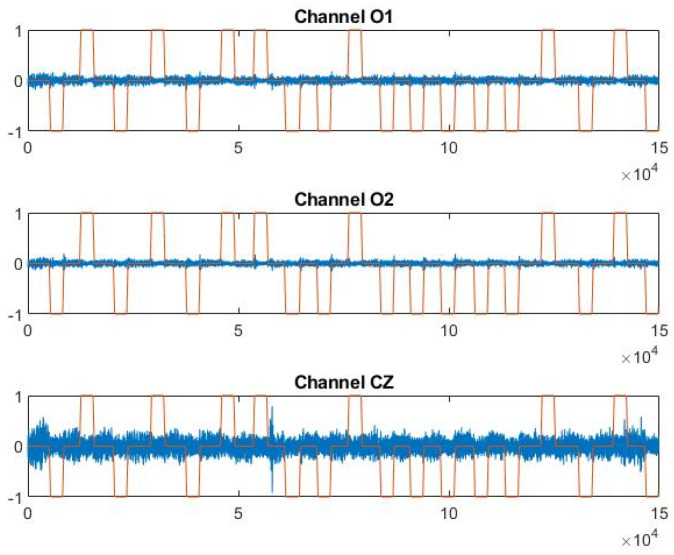
Trend of channels O1, O2, and CZ vs. the output signal y in file P4 F1.

Out of the example, Figure 4 shows P4F1 trend in the three EEG channels versus head movement output signals, filtered and normalized as described in Section 2.3.1.

### 3.2. First Analysis. Identification of the Function f on the First Half File and Verification on the Second Half

Each file was divided into two equals parts; we named the first “training set,” and the second “test set.” The training sets always include samples related to the three possible positions (R, F, L). The results on the testing set can be further classified according to *r* value ranges reported in Table 2 [40].

Table 3 shows the performance indexes on the testing set. In 29 files, only two (P1F8 and P1F10) show a moderate correlation; the others show a strong one instead.

Table 4 and Table 5 report *r* and *MSE* values produced by extracting the functions from the 10 different tests on P1 (rows) and applying them to each test for the same subject (columns). Table 6 and Table 7 report the same data produced from P2 tests.

The cells in the tables are grayed according to the classification given in Table 2 (white = strong correlation; gray = moderate correlation; dark gray = weak correlation).

**Figure 5:**
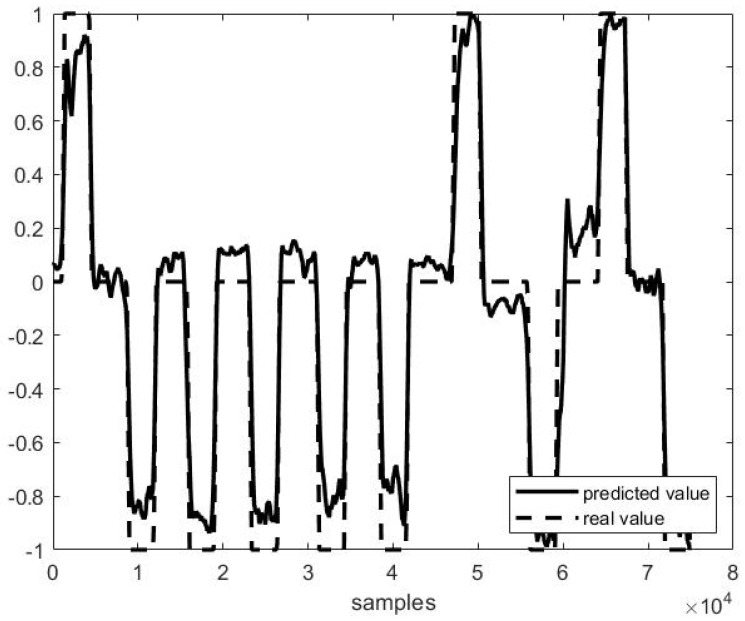
Predicted vs. actual values in P4 F1 testing (*r* = 0.98 and *MSE* = 0.02).

**Figure 6:**
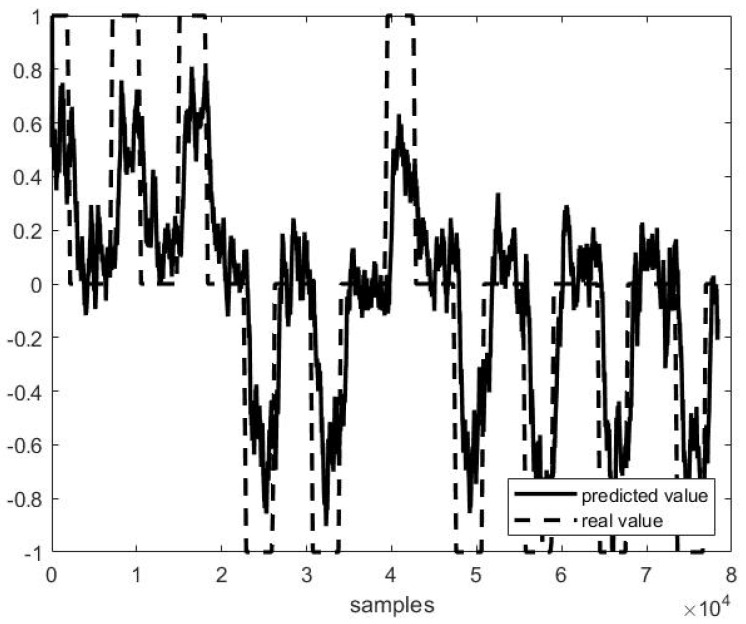
Predicted vs. actual values in the second half of file P3 F1 (*r* = 0.82 and *MSE* = 0.31).

**Figure 7:**
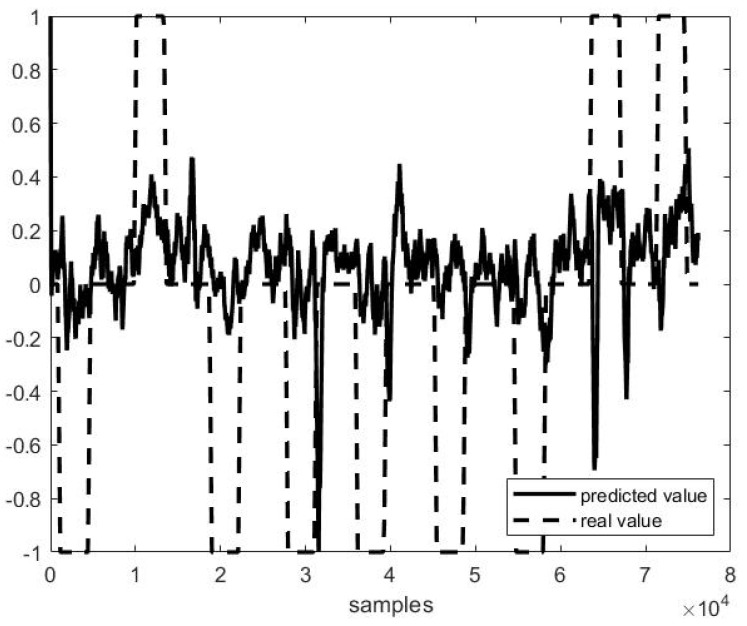
Predicted vs. actual values in P1 F10 testing (*r* = 0.38 and *MSE* = 0.38).

Out of example, Figure 5, Figure 6 and Figure 7 show the trend of three different cases of predictions against the actual head positions.

Figure 5 presents the best case (i.e., on P4F1, *r* = 0.98, Figure 5), and Figure 7 the worst one (i.e., on P1F10, *r* = 0.38, Figure 7), while Figure 6 shows a study case with a medium performance (*r* = 0.82 and *MSE* = 0.31).

### 3.3. Second Analysis. Identification of the Function f on One Participant’s Overall Data and Verification on All Participants’ Overall Data

Following this approach, the files related to the overall experiments for each participant were used to train ANN in order to test the classifier using each function on each test file. Although the function is identified and verified on the same data, the values on the diagonal (see Table 8 and Table 9) showed strong correlation in this analysis too. On the other hand, as expected, testing one subject’s function *f* on another subject’s data returns very low correlation coefficient values, almost close to zero. There is just one case that contradicts this statement: we managed to see that functions coming from P1 return results with a good performance (*r* = 0.52, *MSE* = 0.38) for the P3 case. This exception is surely fortuitous, although it is quite curious noting that P1 is P2′s mother.

## 4. Conclusions

The main contribution of this paper is to address an issue that the literature concerning BCI has paid little attention to: the identification of human head movements (yaw rotation) by EEG signals.

This kind of system is effectively starting to become present in commercial systems at prototypal level. For example, it will be used more and more in the automotive context, with proprietary systems, which will be, however, mostly based on ANN applications. Thus, for the scientific community, it is hard to be completely aware of the current state of the art prototypes. In our opinion, it is important to share experimental results on these subjects.

Concerning the head yaw rotation studied in this work, from the trials performed on ten different participants, spanning more than two hours of experiments, it seems clear that—under some specific limitations—this goal is achievable.

Specifically, after identifying a proper function over a short period of time (a couple of minutes for each participant), this can predict head positions with a quite relevant accuracy for the remaining minutes. Such accuracy is quite relevant (*MSE* < 0.35 and *r* > 0.5, *p* < 0.01) since it was obtained in 26 out of 28 tested files. Once the function is identified for a single file, this generally shows good results on files involving the same participant in the same day.

However, the results obtained in different analyses proved that EEG signals are time variant and the files recorded in a short time interval may be useful to generate a classifier for human head movements following visual stimuli. As a matter of fact, such correlation appears to be time dependent, or more likely, quite susceptible to sensors’ positioning. Besides, a further result of the study, which may represent a drawback but also an important finding of the approach, is related to the fact that the correlation is surely dependent on the specific participant, with the impossibility to predict on another subject when the classifier is trained on another one. This may be a disadvantage in the implementation of the EEG classifier because it seems to be significantly different for each subject, and this precludes the ability to achieve an acceptable level of generalization. However, further studies should demonstrate this when the classifier is identified on a group of several different subjects.

Other important remarks concern the EEG data acquisition reliability, which seems to be extremely dependent on the adherence of the electrodes to the scalp. In the proposed study cases, the two hairless participants achieved better performance in the tests proving that the quality of data collection is closely related to the quality of the predictions.

Future developments will address different arguments. Since, in the trials reported in this paper, EEG is affected both by electrical and illumination stimuli, further efforts should be devoted to separate these two aspects. Secondly, further EEG signal analysis should be performed to outline input-output relations for specific frequency bands.

## Figures and Tables

**Table 1:** The files used to identify the function f.

Part. ID	File ID	Duration Time (s)	Start Time	Head Position Occurrence (Left L, Forward F, Right R)
P1	F1	328	0	L 14.2%, F 59.5%, R 26.3%
P1	F2	310	51 d	L 28.9%, F 60.7%, R 10.5%
P1	F3	319	51 d	L 18.0%, F 59.9%, R 22.1%
P1	F4	336	54 d	L 12.0%, F 60.3%, R 27.7%
P1	F5	335	54 d	L 11.9%, F 60.3%, R 27.8%
P1	F6	328	54 d	L 18.5%, F 60.9%, R 20.6%
P1	F7	306	100 d	L 17.9%, F 61.5%, R 20.6%
P1	F8	307	100 d	L 20.2%, F 59.6%, R 20.2%
P1	F9	328	100 d	L 16.7%, F 59.9%, R 23.4%
P1	F10	305	100 d	L 22.8%, F 61.1%, R 16.1%
P2	F1	341	0	L 21.6%, F 60.7%, R 17.7%
P2	F2	321	68 d	L 19.1%, F 59.8%, R 21.2%
P2	F3	325	68 d	L 12.7%, F 60.3%, R 27.1%
P2	F4	354	68 d	L 22.9%, F 61.7%, R 15.4%
P2	F5	384	68 d	L 17.5%, F 61.3%, R 21.2%
P2	F6	304	85 d	L 11.0%, F 60.4%, R 28.6%
P2	F7	314	85 d	L 17.0%, F 59.5%, R 23.5%
P2	F8	312	85 d	L 30.5%, F 60.9%, R 8.7%
P2	F9	316	85 d	L 23.3%, F 62.0%, R 14.7%
P2	F10	316	85 d	L 17.0%, F 60.1%, R 22.9%
P3	F1	314	0	L 19.1%, F 59.6%, R 21.3%
P4	F1	300	0	L 25.9%, F 59.3%, R 14.8%
P5	F1	399	0	L 16.8%, F 60.0%, R 23.2%
P6	F1	308	0	L 11.0%, F 60.7%, R 28.4%
P7	F1	356	0	L 23.0%, F 61.7%, R 15.8%
P8	F1	304	0	L 25.5%, F 61.7%, R 12.8%
P9	F1	366	0	L 19.7%, F 60.4%, R 19.9%
P10	F1	377	0	L 24.8%, F 59.6%, R 15.6%
P10	F2	339	1 h	L 22.1%, F 59.9%, R 18.0%

**Table 2:** Threshold values to evaluate correlation performance.

r	Correlation Performance
0.50 ≤ *r* ≤ 1	strong
0.30 ≤ *r* < 0.50	moderate
*r* < 0.30	Weak

**Table 3:** Prediction performances by the first analysis.

Participant ID	File ID	*MSE*	*r*
P1	F1	0.12	0.86
P1	F2	0.20	0.80
P1	F3	0.32	0.78
P1	F4	0.14	0.84
P1	F5	0.26	0.78
P1	F6	0.21	0.71
P1	F7	0.30	0.61
P1	F8	0.37	0.48
P1	F9	0.42	0.79
P1	F10	0.38	0.38
P2	F1	0.31	0.71
P2	F2	0.19	0.86
P2	F3	0.12	0.88
P2	F4	0.16	0.86
P2	F5	0.16	0.82
P2	F6	0.29	0.82
P2	F7	0.30	0.78
P2	F8	0.28	0.57
P2	F9	0.27	0.76
P2	F10	0.35	0.87
P3	F1	0.31	0.82
P4	F1	0.02	0.98
P5	F1	0.13	0.91
P6	F1	0.37	0.59
P7	F1	0.35	0.66
P8	F1	0.33	0.76
P9	F1	0.33	0.78
P10	F1	0.18	0.89
P10	F2	0.32	0.93

**Table 4:** *r* Values (P1).

*r*	F1	F2	F3	F4	F5	F6	F7	F8	F9	F10
F1	0.90	−0.18	−0.21	−0.73	−0.39	−0.74	0.40	−0.17	−0.35	−0.15
F2	−0.23	0.78	0.59	−0.79	−0.70	−0.78	−0.32	−0.21	−0.42	−0.47
F3	−0.33	0.52	0.71	−0.8	−0.65	−0.81	−0.19	−0.35	−0.10	−0.14
F4	0.01	−0.59	−0.56	0.84	0.73	0.82	0.04	0.57	0.15	0.19
F5	0.06	−0.53	−0.5	0.84	0.82	0.84	0.49	−0.09	−0.01	0.08
F6	0.17	−0.54	−0.47	0.84	0.76	0.85	0.37	−0.15	0.13	0.13
F7	0.70	−0.14	−0.14	0.70	0.60	0.70	0.67	0.27	−0.07	−0.02
F8	−0.11	−0.48	−0.40	0.80	0.68	0.81	0.35	0.55	0.31	0.08
F9	−0.69	−0.06	0.22	0.41	0.33	0.45	0.39	0.07	0.80	0.68
F10	−0.66	−0.32	0.00	0.78	0.52	0.77	0.20	0.31	0.83	0.79

**Table 5:** *MSE* Values (P1).

*MSE*	F1	F2	F3	F4	F5	F6	F7	F8	F9	F10
F1	0.09	0.51	0.41	0.52	0.49	0.47	0.35	0.41	0.40	0.40
F2	0.54	0.17	0.46	0.93	1.23	1.33	0.86	0.78	0.78	0.68
F3	0.48	0.41	0.31	1.10	1.72	1.73	0.50	0.41	0.40	0.38
F4	1.25	2.64	2.18	0.11	0.19	0.13	2.39	2.46	2.26	2.79
F5	0.79	1.88	1.41	0.14	0.13	0.13	1.95	1.84	1.50	1.93
F6	1.05	2.13	1.64	0.12	0.17	0.11	2.25	2.11	1.70	2.16
F7	0.28	0.43	0.40	0.58	0.47	0.45	0.25	0.38	0.40	0.38
F8	0.48	0.47	0.42	1.01	1.08	1.07	0.33	0.30	0.37	0.40
F9	0.85	0.45	0.37	0.34	0.35	0.35	0.34	0.41	0.32	0.35
F10	0.88	0.42	0.43	0.55	0.71	0.51	0.36	0.38	0.35	0.34

**Table 6:** *r* Values (P2).

*r*	F1	F2	F3	F4	F5	F6	F7	F8	F9	F10
F1	0.78	0.62	0.77	0.62	0.77	0.41	−0.01	0.01	0.02	0.0
F2	0.09	0.86	0.86	0.86	0.85	0.29	0.51	0.61	0.70	0.52
F3	0.01	0.77	0.87	0.77	0.84	0.21	0.31	0.11	0.26	0.33
F4	0.00	0.78	0.86	0.76	0.86	0.27	0.37	0.38	0.42	0.43
F5	−0.14	0.83	0.86	0.83	0.87	0.30	0.60	0.58	0.72	0.60
F6	0.15	0.42	0.60	0.42	0.83	0.75	0.70	0.69	0.77	0.80
F7	−0.00	−0.12	0.44	−0.12	0.39	0.42	0.80	0.72	0.84	0.86
F8	0.15	−0.22	−0.24	−0.22	−0.11	0.37	0.71	0.72	0.8	0.65
F9	0.16	−0.20	−0.15	−0.20	−0.21	0.53	0.62	0.66	0.81	0.72
F10	−0.11	−0.36	0.05	−0.36	0.04	0.41	0.78	0.71	0.83	0.87

**Table 7:** *MSE* Values (P2).

*MSE*	F1	F2	F3	F4	F5	F6	F7	F8	F9	F10
F1	0.29	0.26	0.29	0.43	0.31	0.31	0.63	1.13	0.93	0.68
F2	0.40	0.16	0.15	0.17	0.15	0.51	0.47	0.30	0.36	0.46
F3	0.43	0.20	0.13	0.18	0.16	0.40	0.44	0.34	0.35	0.45
F4	0.44	0.17	0.15	0.15	0.54	0.58	0.33	0.43	0.59	0.21
F5	0.42	0.20	0.15	0.16	0.15	0.48	0.51	0.31	0.40	0.51
F6	0.40	0.34	0.28	0.32	0.29	0.25	0.36	0.52	0.41	0.35
F7	0.38	0.42	0.38	0.33	0.36	0.36	0.29	0.33	0.28	0.31
F8	0.39	0.55	0.57	0.41	0.47	0.47	0.39	0.26	0.30	0.39
F9	0.37	0.55	0.50	0.39	0.44	0.37	0.35	0.27	0.26	0.34
F10	0.39	0.44	0.40	0.36	0.38	0.32	0.30	0.34	0.28	0.30

**Table 8:** *r* values in the second analysis.

*r*	P1	P2	P3	P4	P5	P6	P7	P8	P10	P10
P1	0.41	−0.09	0.08	−0.07	−0.02	0.21	0.01	0.03	−0.07	0.01
P2	−0.36	0.64	−0.17	0.06	0.19	−0.30	−0.09	−0.02	0.14	0.23
P3	0.52	−0.53	0.81	0.07	−0.26	0.60	−0.22	0.41	−0.05	0.08
P4	−0.07	−0.17	−0.42	0.93	0.08	−0.48	−0.72	−0.24	−0.04	0.19
P5	−0.31	−0.02	−0.50	−0.62	0.90	−0.27	0.80	−0.64	−0.15	0.02
P6	−0.04	−0.21	0.45	0.10	0.11	0.53	−0.56	−0.48	−0.04	0.03
P7	0.31	−0.08	−0.52	−0.16	0.33	−0.57	0.67	−0.11	−0.27	0.23
P8	0.51	−0.32	0.67	0.43	−0.62	−0.57	0.41	0.72	−0.09	0.10
P9	−0.06	0.78	0.19	0.42	0.40	0.11	−0.26	0.01	0.80	−0.59
P10	−0.63	−0.50	0.29	−0.33	−0.58	−0.19	0.62	−0.23	−0.74	0.84

**Table 9:** *MSE* values in the second analysis.

*MSE*	P1	P2	P3	P4	P5	P6	P7	P8	P9	P10
P1	0.33	0.41	0.46	0.40	0.59	0.41	0.45	0.41	0.46	0.58
P2	0.55	0.27	0.50	0.39	0.71	0.43	0.42	0.49	0.44	0.44
P3	0.38	0.41	0.25	0.40	0.96	0.36	0.43	0.42	0.47	0.63
P4	0.74	2.26	2.40	0.05	6.95	0.62	0.67	0.70	1.01	0.61
P5	0.40	0.40	0.65	0.42	0.12	0.45	0.27	0.89	0.47	0.62
P6	0.37	0.40	0.36	0.40	1.18	0.33	0.42	0.49	0.38	0.78
P7	0.37	0.37	0.42	0.37	0.69	0.42	0.32	0.39	0.43	0.46
P8	0.33	0.37	0.43	0.35	0.49	0.62	0.32	0.28	0.45	0.44
P9	0.75	0.64	0.72	0.72	0.68	0.85	0.81	0.96	1.39	0.34
P10	0.63	0.63	0.52	0.60	0.85	0.60	0.51	0.61	0.20	0.96

## Data Availability

The data presented in this study are available on request from the corresponding author. The data are not publicly available due to restrictions present in the informed consent.
